# A comprehensive computer aided approach to design of earthen dams of dry flood-control reservoirs

**DOI:** 10.1016/j.mex.2023.102077

**Published:** 2023-02-14

**Authors:** Maciej Sobótka, Adrian Różański, Jakub Rainer, Mikołaj Masłowski

**Affiliations:** Wrocław University of Science and Technology, Faculty of Civil Engineering, Wybrzeże Wyspiańskiego 27, 50-370 Wrocław, Poland

**Keywords:** Hydrotechnical structure, Flood protection, Geotechnics, Numerical analysis, Hydromechanical coupling, Computer aided design of earthen dams of dry flood-control reservoirs

## Abstract

A generalized, comprehensive approach to geotechnical design of earthen dams of dry flood-control reservoirs is developed. It consists of three steps:

• In Step 1 three-dimensional (3D) model of terrain and dam subsoil is created. The arrangement of geotechnical layers is reconstructed based on results of geotechnical investigation using geostatistical interpolation, namely, kriging.
• Step 2 involves 3D finite element (FE) analysis of transient groundwater flow through the subsoil. Anti-filtration barrier extent is determined by appropriate parametric analysis and the condition of critical hydraulic gradients is verified.
• Step 3 concerns deformation assessment and stability analysis. Computations are carried out for selected cross-sections of the dam. At this stage transient coupled problem of deformation and groundwater flow is considered. Stability is assessed with the use of shear strength reduction technique.

The proposed approach enables verification of all relevant conditions of limit states. Furthermore, it has been developed in such a way as to enable computationally efficient analysis of considered phenomena. The method constitutes a general framework of design procedure. It can be easily adapted to conform any standard requirements, e.g., by taking adequate values of partial safety factors. Moreover, design solutions can be optimized through parametric analyzes.

## Graphical abstract

**Figure:**
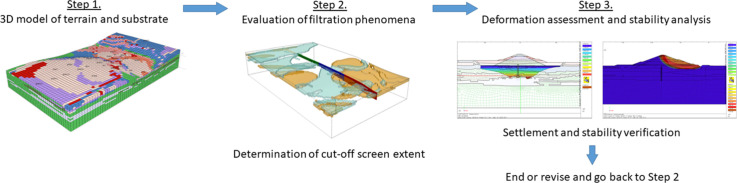

Specifications table

**Table:** 

Subject area:	Environmental Science
More specific subject area:	Geology and Hydrology
Name of your method:	Computer aided design of earthen dams of dry flood-control reservoirs
Name and reference of original method:	D. Łydżba, A. Różański, M. Sobótka, M. Pachnicz, S., Grosel, J. Rainer, A comprehensive approach to the optimization of design solutions for dry anti-flood reservoir dams. Stud. Geotech. Mech. 43(3) (2021) 270–284.
Resource availability:	https://doi.org/10.2478/sgem-2021-0016

**Method details**

## Overview

Dry flood-control reservoirs are important elements of passive flood protection infrastructure. Such reservoirs remain empty most of the time and fill up during high water [1]. This way, the outflow from the reservoir is limited so that to keep the downstream areas safe. To ensure proper and safe operation of such facilities, they must be properly designed. The design method presented in the article concerns geotechnical aspects and applies in particular to earthen dams of flood-control reservoirs. From technical point of view the most important issue is to ensure the stability of dam and its substrate with regard to mechanical strength as well as phenomena related to groundwater flow. A significant difficulty in designing this type of structures is the fact that they temporarily dam up water. This entails transient states of groundwater flow. As a further consequence mechanical stability (in terms of factor of safety) changes over time in the course of flood detention. Moreover, the water filtration in the substrate is intrinsically three dimensional (3D) and this should be taken into account in the design procedure. Thus, the proposed method is based on creation of 3D models followed by appropriate numerical computations in order to verify the stability of the dam and conditions of safe water flow in the substrate. Originally, the method [2] has been developed so that to fulfill requirements given in Eurocode 7. Nevertheless, it can be easily adapted to conform any standard mostly by taking into account the appropriate safety factors. The presented method has been formulated in such a way as to allow credible analysis of the phenomena occurring within the dam and its substrate. It assumes using numerical methods, most preferably finite element (FE) analysis, to obtain solutions of initial-boundary value problems. They set the basis for verification of appropriate limit states. The procedure assumes rational way of using numerical models with minimal use of resources to reduce the computation time. Simplifications of the model are proposed in order to reduce the 3D computational domain of groundwater flow analysis. Mechanical stability is assessed using shear strength reduction (SSR) technique. Since it requires application of hydromechanical coupling which introduces significant complexity to the model the task is implemented for extracted cross-sections as 2D problems. Furthermore, the proposed framework of computation not only enables verification of appropriate conditions of limit states, but also adjustment of design assumptions, e.g., an extent of the cut-off screen. The method is illustrated with examples of its application for two designed recently dry flood-control reservoirs located in south-western Poland, i.e. Szalejów Górny [3] and Krosnowice [4]. Computations for the mentioned objects were carried out using ZSoil finite element method code. However, the use of proposed method is not restricted to a particular type of software.

*Step 1. Reconstruction of the terrain surface and probable soil layers arrangement*

**Fig. 1:**
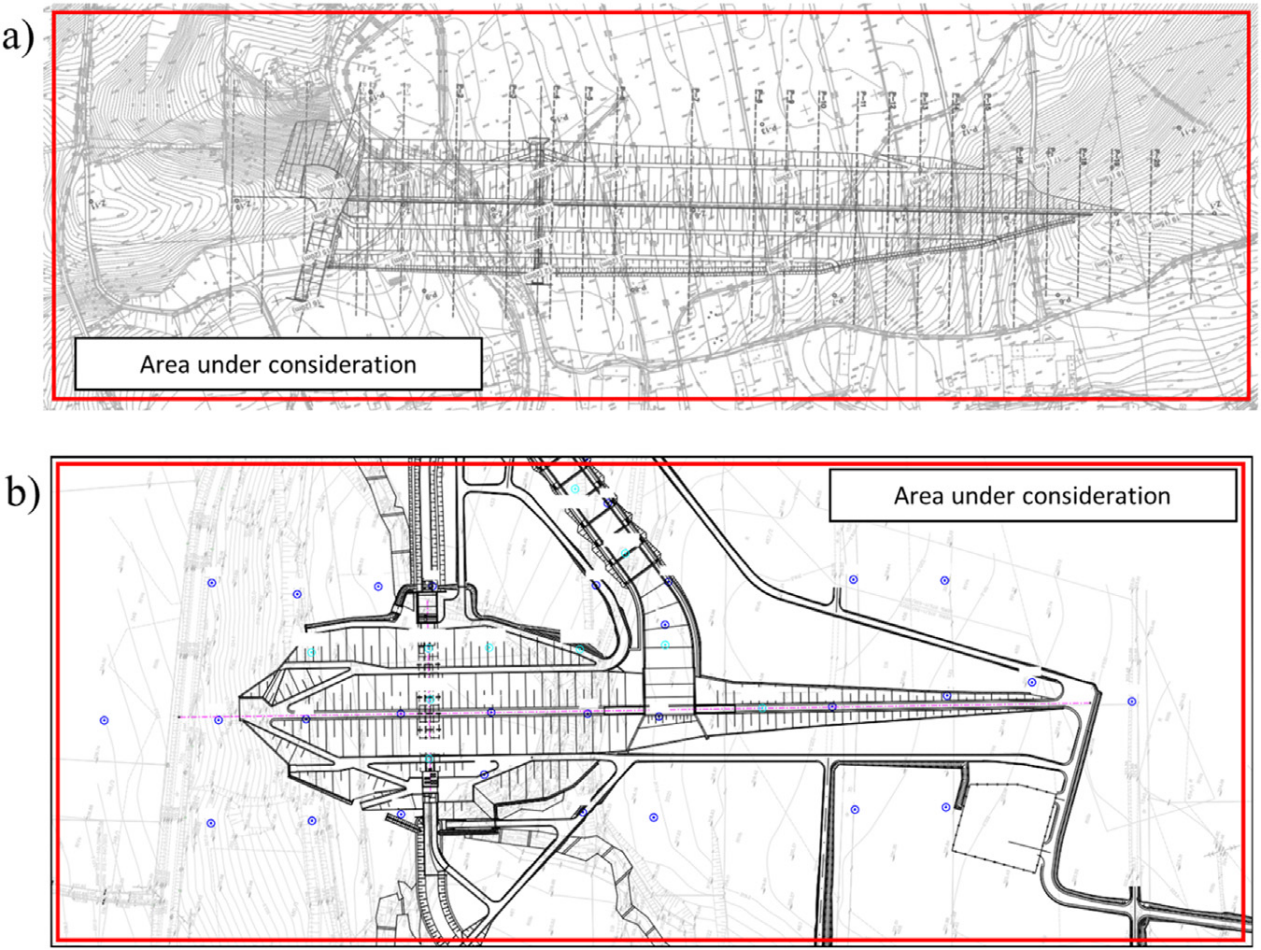
Specified areas under consideration: a) Szalejów Górny, b) Krosnowice.

Designing a dam for an earthen flood-control reservoir requires a sequence of computations performed on a three-dimensional numerical model. However, the creation of such a model must be preceded by specifying the area covered by the analysis, mapping the actual surface of the terrain and reconstructing the probable arrangement of geotechnical layers. These three tasks are carried out on the basis of the available design documentation containing a hypsometric map of the terrain and results of geotechnical investigation, e.g. boreholes or soundings. The domain should cover at least the area of the dam. However, to minimize the influence of boundary conditions on the results of numerical calculations, it is strongly recommended to extend the domain by at least the total width of the dam in each direction (Fig. 1).

**Fig. 2:**
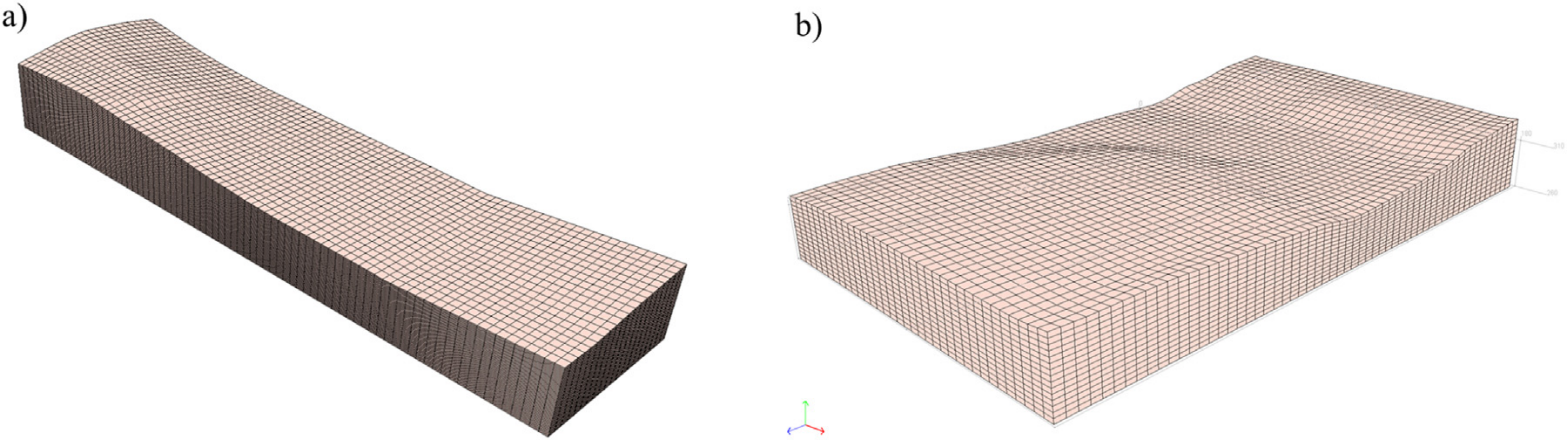
Preliminary FE computational domain created in ZSoil software: a) Szalejów Górny, b) Krosnowice

A three-dimensional terrain mesh model can be generated using e.g. CAD tools. It is then transferred into the FE software (where the numerical simulations are performed) and creates the top boundary of the 3D computational domain. The model must also be bounded from below, and it is important here that the depth of the subsoil should be at least several times greater than the height of the dam. The exemplary preliminary models of the terrain and subsoil, together with the division into finite elements are shown in Fig. 2.

**Fig. 3:**
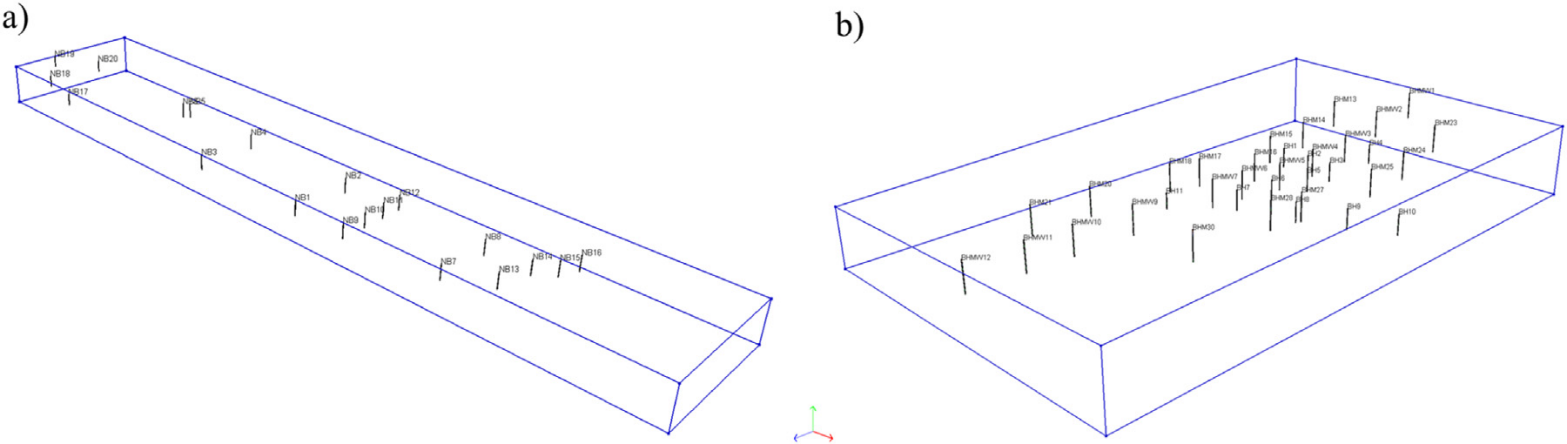
Location of available boreholes and boundary of the computational domain: a) Szalejów Górny, b) Krosnowice.

The final numerical model is created by supplementing the preliminary one (Fig. 2) with the reconstructed system of geotechnical layers. In practice, the reconstruction consists in assigning the appropriate material and its parameters to each element. This task can be performed with the use of various techniques, however, geostatistical interpolation, e.g. kriging [5], seems to be the most rational here. The interpolation treats the elevation of layer bottom and ceiling as a random variable [6,7]. Its values are fixed in the locations of available boreholes and interpolated in the entire domain. The number of available boreholes depends on the implemented subsoil investigation program. It is obvious that the more information about the subsoil is available, the better the reconstruction of the actual layer arrangement under the dam. Usually, for economic reasons, the number of boreholes or soundings is limited to the minimum requirements in accordance with applicable standards and regulations. In Fig. 3 available boreholes (treated as fixed data within kriging) for considered dams are presented.

**Fig. 4:**
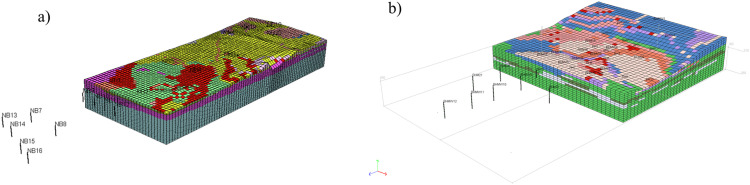
Numerical models of terrain and subsoil showing the result of kriging: a) Szalejów Górny, b) Krosnowice.

Interpolation by kriging requires the assumption of the so-called semi-variogram function which measures “the strength” of statistical correlation as a function of distance. In this regard, the spherical and exponential models are most often used [8]. An important parameter to be established is also the correlation length/radius [6]. This can be evaluated by fitting models to sample semi-variograms by weighted least squares approximation or by assuming value of correlation length in relation to the dimensions of the dam [8,9]. Utilizing the given data provided by available boreholes (Fig. 3) the process of interpolation consists in minimizing the error variance of the interpolated variable. Fig. 4 presents numerical models with reconstruction of soil layers spatial arrangement obtained with spherical models and assumption that correlation length is equal to 500 m. The result of kriging is shown in part of the volume in the back, while in the foreground only the location of boreholes is presented.

*Step 2. Evaluation of groundwater flow and determination of cut-off screen depth*

Creation of the numerical model as described in Step 1 is followed by groundwater flow assessment. In particular, cut-off screen depth and its anchorage length in the abutments are determined by a proper parametric analysis. The extent of anti-filtration barrier must be enough to meet the conditions described next. The main consideration in this context is the limit state related to unfavorable phenomena resulting from groundwater flow in the subsoil such as internal erosion, soil boiling or soil uplift. The conditions of soil boiling or uplift occurrence are verified during the numerical analysis of the overall dam stability (see Step 3). To avoid possible occurrence of internal erosion, i.e. suffosion, the critical filtration gradients must not be exceeded. It is expressed by the following formula:

(1) $$\gamma_{i}\cdot i\leq i_{cr}$$

where:

*i* – hydraulic gradient,

*i_cr_* – critical values of hydraulic gradient for a given soil,

*γ_i_* – safety factor determined according to national regulations and/or relevant standards.

**Fig. 5:**
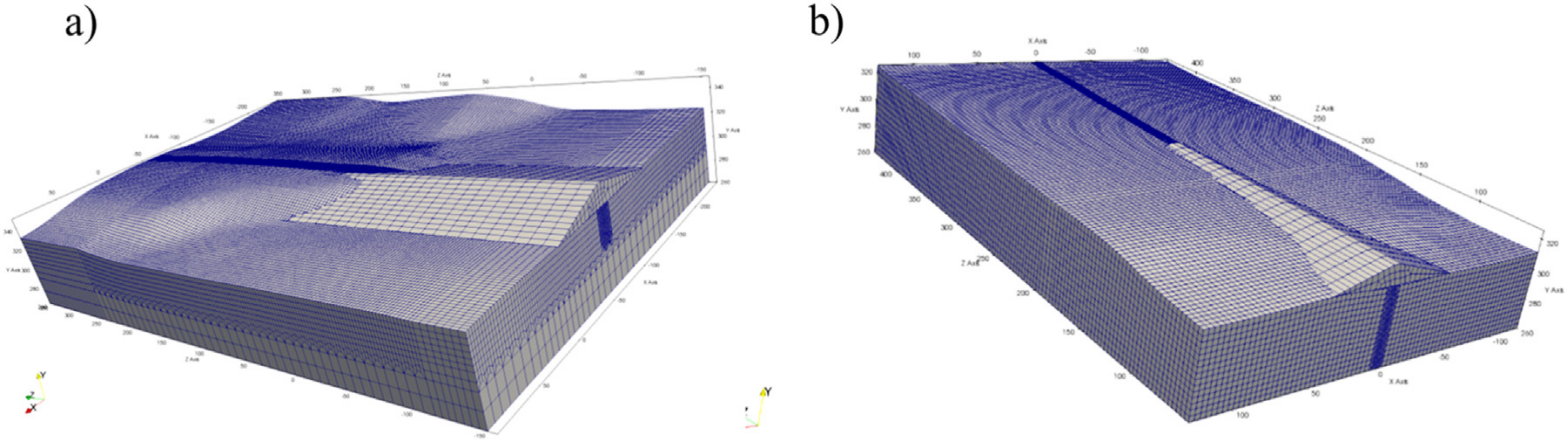
Computational models for groundwater flow analysis: a) Szalejów Górny, b) Krosnowice.

In order to verify condition (1), first an initial-boundary value problem concerning transient flow in partially saturated medium has to be solved. In this regard, commercial software most often utilize van Genuchten model of soil-water retention curve [10]. Furthermore, Richards hypothesis of gas phase continuity in the aeration zone allows one to perform single phase flow analysis. Such an approach is recommended in the proposed method. The computations are performed using the 3D model resulting from Step 1. For saving computational resources the entire model can be divided into two parts. Then, each of two abutments are considered separately. This routine is optional. However, it was utilized in the examples illustrating the method. Appropriate models are depicted in Fig. 5.

**Fig. 6:**
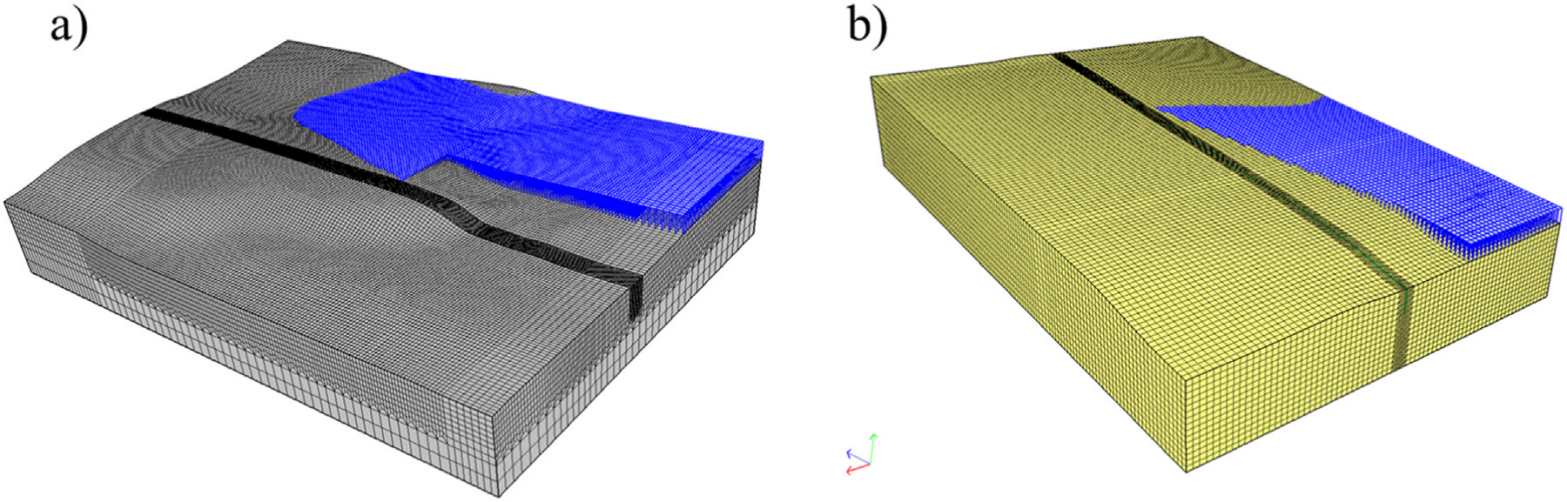
Water pressure boundary conditions in the basin: a) Szalejów Górny, b) Krosnowice.

For a given model domain boundary conditions (BCs) are to be defined. The groundwater table level known from geotechnical investigation is extrapolated to the vertical planes limiting the model domain on upstream and downstream sides. The relevant BCs should be set as pore water pressure linearly increasing with depth. At the level of water table the value of pore pressure is set to 0 and a constant gradient of the pore pressure along elevation ordinate corresponds to the hydrostatic increase. Remaining parts of model boundary are at first set to be impermeable. For boundary conditions set in this manner the steady state problem of groundwater flow is solved. Obtained distribution of pore pressure is set as initial condition for the following part of computation involving analysis of transient filtration flow. In order to perform such analysis a new boundary condition is to be defined on the part of a boundary which corresponds to the basin of the reservoir. This BC represents hydrostatic water pressure exerted on the basin in the course of flood wave attenuation. This BC is depicted in Fig. 6. The distribution of actual water pressure set as the boundary condition results from the anticipated, time-varying damming level in the reservoir. In general the function of water table level in the reservoir versus time is calculated based on the basin geometry and stream discharge function assumed for the design.

The distribution of the hydraulic gradient *i* is determined according to Darcy's law based on the fluid velocity distributions obtained from FE analysis. Within the saturation zone the following formula applies:

(2) $$i=\frac{v}{k},$$

where:

k− hydraulic conductivity of soil,

v− fluid velocity.

The critical values of hydraulic gradients *i_cr_* in different soil types are determined most often from empirical relationships. Among the most widespread there are the formulas of Sichardt [11] and Abramov [12], with the latter being more conservative. Eventually, the Abramov formula is proposed to be applied. In terms of critical gradient it expresses as:

(3) $$i_{cr}=0.032\cdot k^{-\frac{2}{3}},$$

where *k* is given in m/s.

It is inconvenient to verify condition (1) using its direct form because the critical values of the hydraulic gradient are generally different for each layer. For this reason, it is proposed to transform the condition (1) to the following form [1]:

(4) $$F_{i}=\frac{\gamma_{i}\cdot i}{i_{cr}}\leq 1.$$

**Fig. 7:**
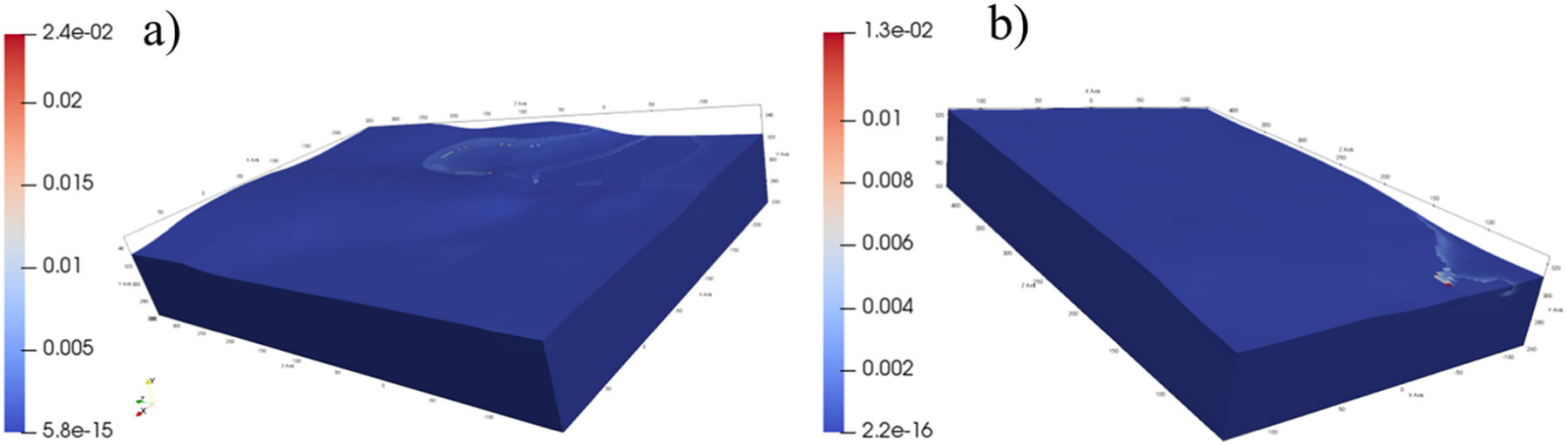
Distribution of *F_i_* parameter: a) Szalejów Górny, b) Krosnowice.

The parameter *F_i_* is the multiplicity of the limit admissible gradient. Then, the interpretation of *F_i_* values can be applied to the entire domain: if *F_i_* ≤ 1.0 then the condition of permissible hydraulic gradient value is met. On the other hand, if *F_i_* >1.0 in any area of the substrate, internal erosion could potentially occur. For transient flow considered the parameter *F_i_* can vary with time. Thus its maximum value must be searched for among analyzed time steps. The examples of *F_i_* distributions are presented in Fig. 7. These particular examples show that the maximum values of *F_i_* (over time and space) are much less than 1. It means the condition (4) is met and, thus, the risk of suffusion occurrence is of no concern.

It is to be noted that in general the condition (3) locally can be violated without indicating dangerous water flow states. This situation may take place at the base of the cut-off wall due to the singularity of numerical solution next to the "sharp edge" of subdomain. In addition, minor, local violations of condition (3) may be conditionally accepted as long as they are below the ground surface, or next to it with flow velocity vector directed to the ground.

**Fig. 8:**
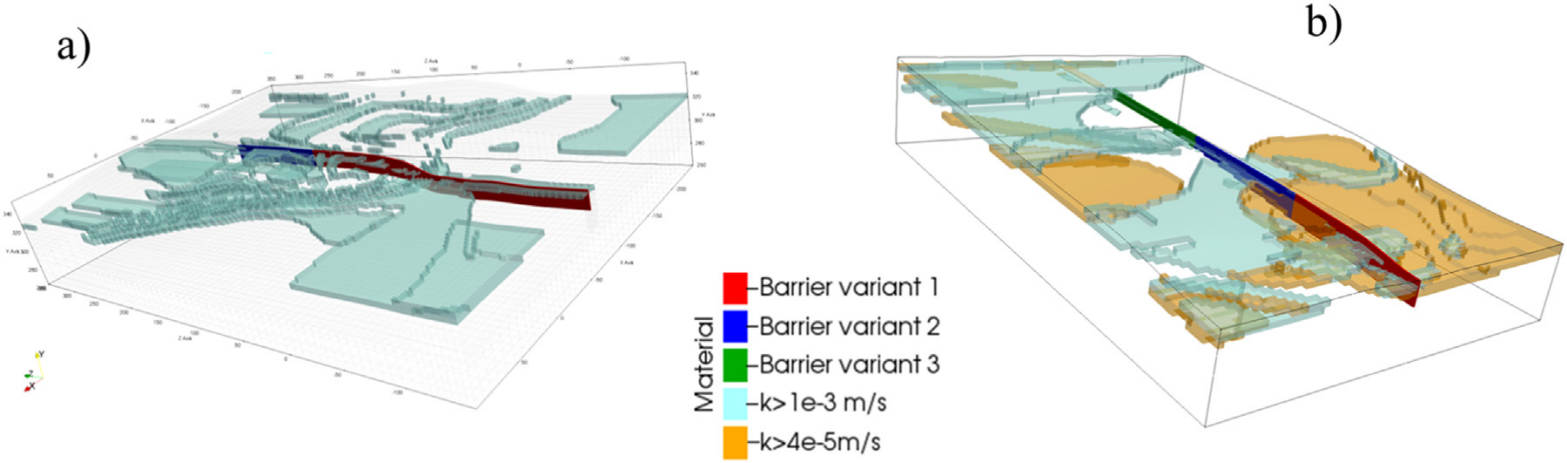
Perspective view of permeable layers: a) Szalejów Górny, b) Krosnowice.

Furthermore, it is advised to place the cut-off wall so that to partition permeable layers to limit the filtration flow under the dam as well as through abutments. It is convenient to visualize the permeable layers in 3D model to inspect whether they are split with the anti-filtration barrier. Such views of the model are presented in Fig. 8. The particular layer is considered permeable if its hydraulic conductivity *k*>1.0e-3 m/s (see blue layers in Fig. 8) or it is at least two orders of magnitude larger than hydraulic conductivity of a dominant layer in the subsoil at the level of cut-off wall base. Such a case is marked orange in Fig. 8b. If permeable layers cover larger areas of the subsoil then the extent of cut-off wall should be determined in parametric analysis by testing different depths and anchorage lengths and checking whether the condition (3) is met. In this context, please note considered variants of cut-off wall anchorage lengths marked with different colors in Fig. 8.

Above-described requisites regarding groundwater flow states are referred to as necessary conditions. These must be considered in conjunction with the further requirements described in the next step. Moreover, it may also be necessary to carry out steady-state flow analysis for maximum damming level assumed in the design. It may be required due to the national regulations or standards. Such additional analysis also makes sense if a transformation of the object into a reservoir with constant water level is considered as an option for the future operation scheme.

*Step 3. Deformation assessment and stability analysis*

**Fig. 9:**
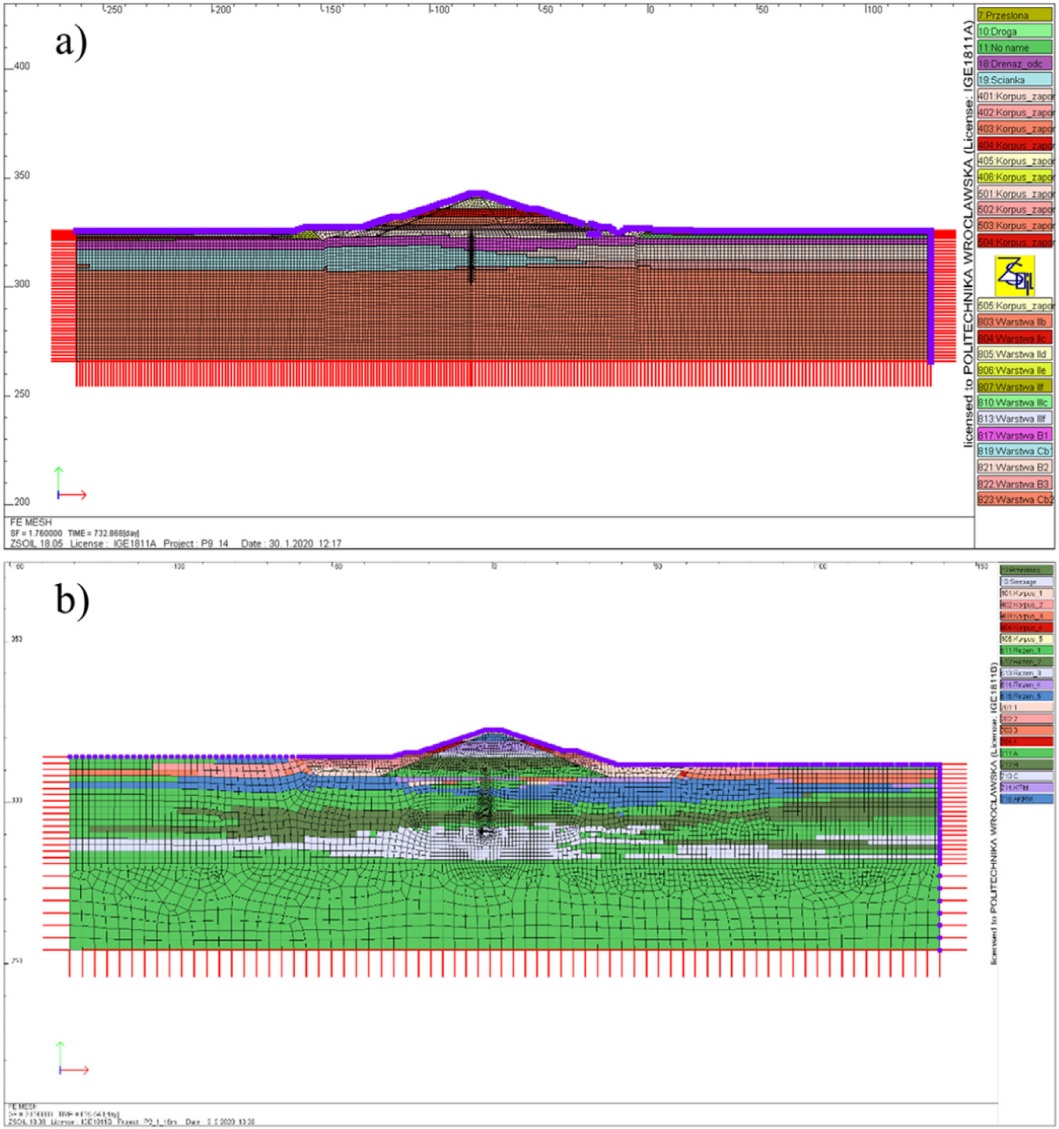
FE numerical model created in ZSoil software: a) Szalejów Górny, b) Krosnowice.

**Fig. 10:**
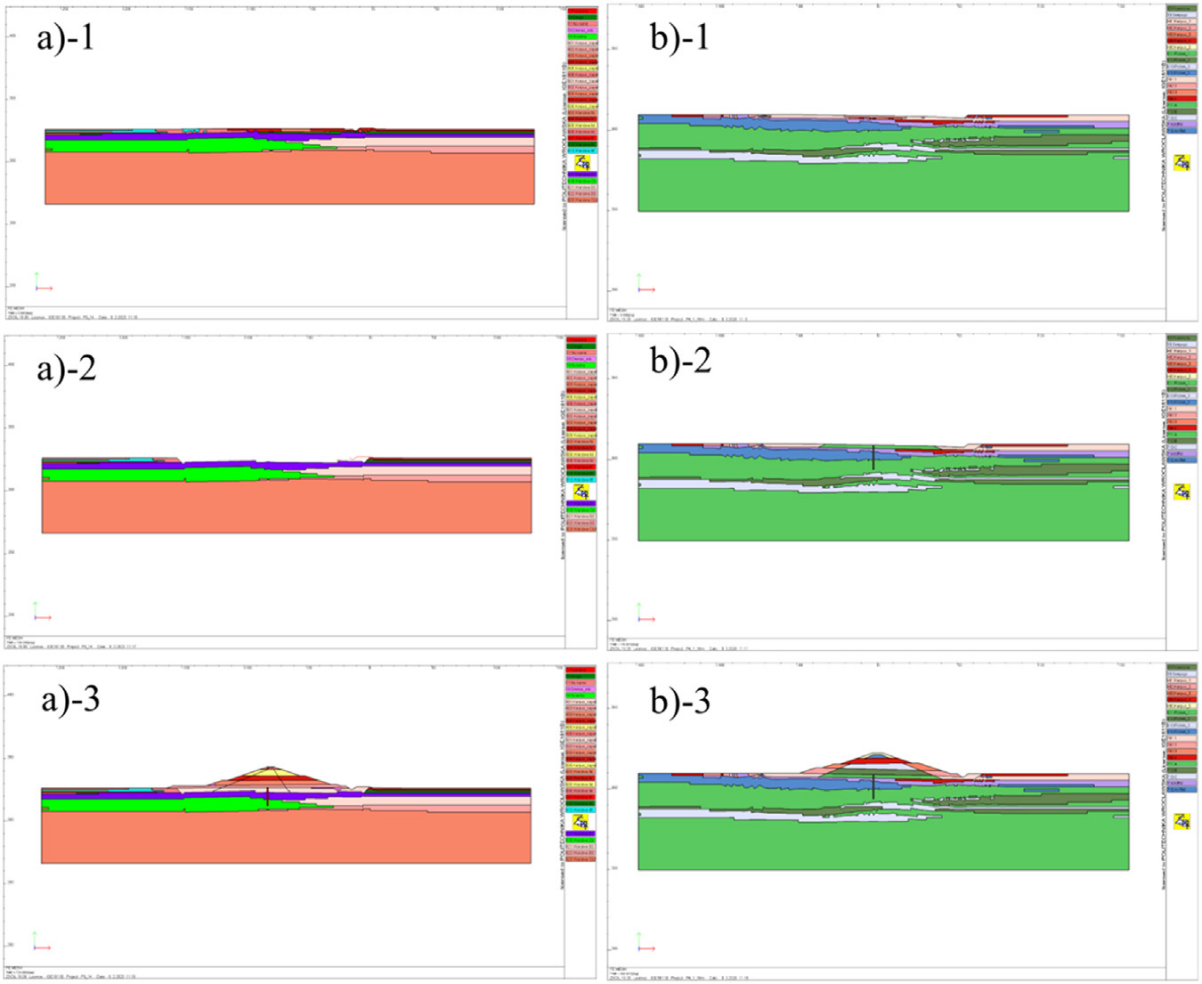
Numerical models of dams at selected stages: a) Szalejów Górny, b) Krosnowice; 1 – starting geometry for the initial state, 2 – execution of anti-filtration barrier after excavation, 3 – dam construction completed.

Analyses within the scope of Step 3 for the assessment of deformation and settlement of subsoil, as well as stability analyses (using the SSR method) [13,14] are carried out for selected plane cross-sections of the dam. Their domains can be extracted as the cross-sections of the three-dimensional model described in Step 1. The number of cross-sections and their location is to be determined according to the size of the dam, the arrangement of the soil layers and the geometry of the terrain. It should be noted that the stability results obtained from a 2D analysis are generally more conservative than from a 3D analysis [15]. Therefore such an approach establishes a safe estimate. The elastic-plastic constitutive relations must be assumed for the subsoil and dam body. The Mohr-Coulomb model is adopted in this regard. Furthermore staged construction of the dam is taken into account. In other words, erection of the dam is modeled as a stepwise (sequential) ”extension” of the model geometry by successively "appearing" layers. Figs 9 and 10 show examples of FE numerical models of dam cross-sections and selected construction stages included in the analyses.

**Fig. 11:**
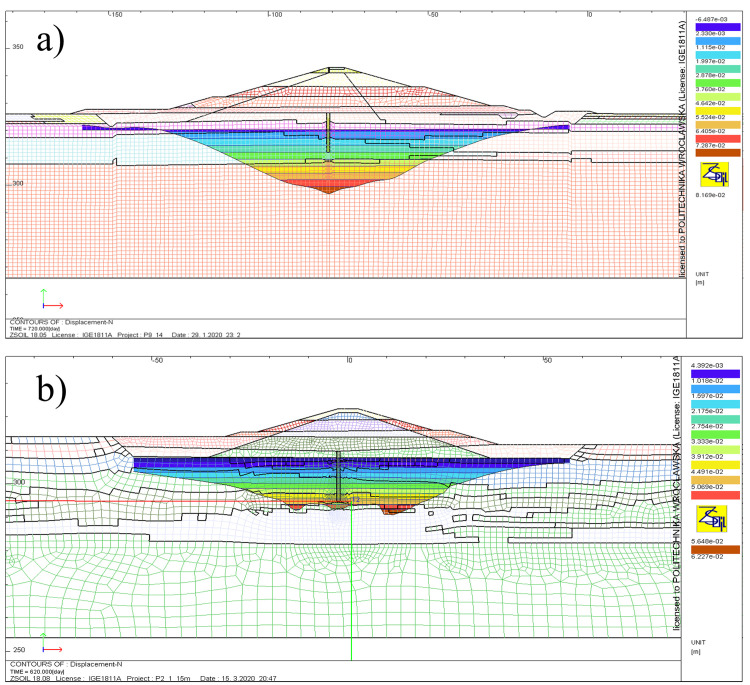
Settlements at the foundation level resulting from the construction of dam: a) Szalejów Górny, b) Krosnowice.

Settlements resulting from the construction of dam are computed as vertical displacements at the foundation level. Unless it is automated in the particular software used the settlement is to be calculated as the increment of vertical displacement between reference state (before dam construction) and the one corresponding to the completion of the structure. Examples of calculated settlement are presented in Fig. 11. With regard to the settlements calculated in this way, the condition of the appropriate limit state is met if the maximum value of obtained displacement is not greater than maximum admissible value. The latter is given in the relevant standard or legal requirements applicable in a given country.

The computations of dam subsoil deformation as well as stability assessment is carried out taking into account the hydromechanical coupling. This entails a significant increase in computation time compared to uncoupled analysis described in Step 2. This is the main reason for performing deformation and stability analyses as two-dimensional problems within the proposed design approach. On the other hand, using so called effective parameters of strength and considering coupled problem enables to verify a few limit states at ones. It includes general stability as well as filtration related phenomena that may occur, i.e. soil boiling and soil uplift mentioned earlier. All these limit states require the same condition to be met. The factor of safety (*FOS*) has to be not less than *FOS*_min_. The latter is minimum required value of *FOS* provided by relevant standard or legal requirement. Regardless of particular value of *FOS*_min_ (and any partial safety factors) it can be convenient to represent condition of stability in the form:

(5) $$F\left(t\right)=\frac{FOS}{FOS_{min}}\geq 1,$$

where *F*(*t*) stands for relative safety margin.

**Fig. 12:**
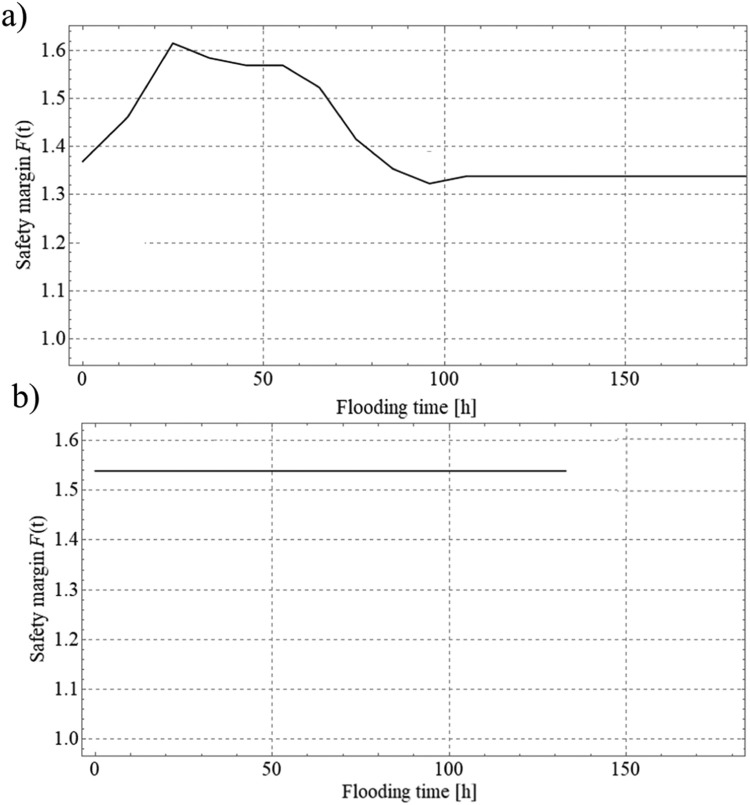
Variation of the relative safety margin in time: a) Szalejów Górny, b) Krosnowice.

**Fig. 13:**
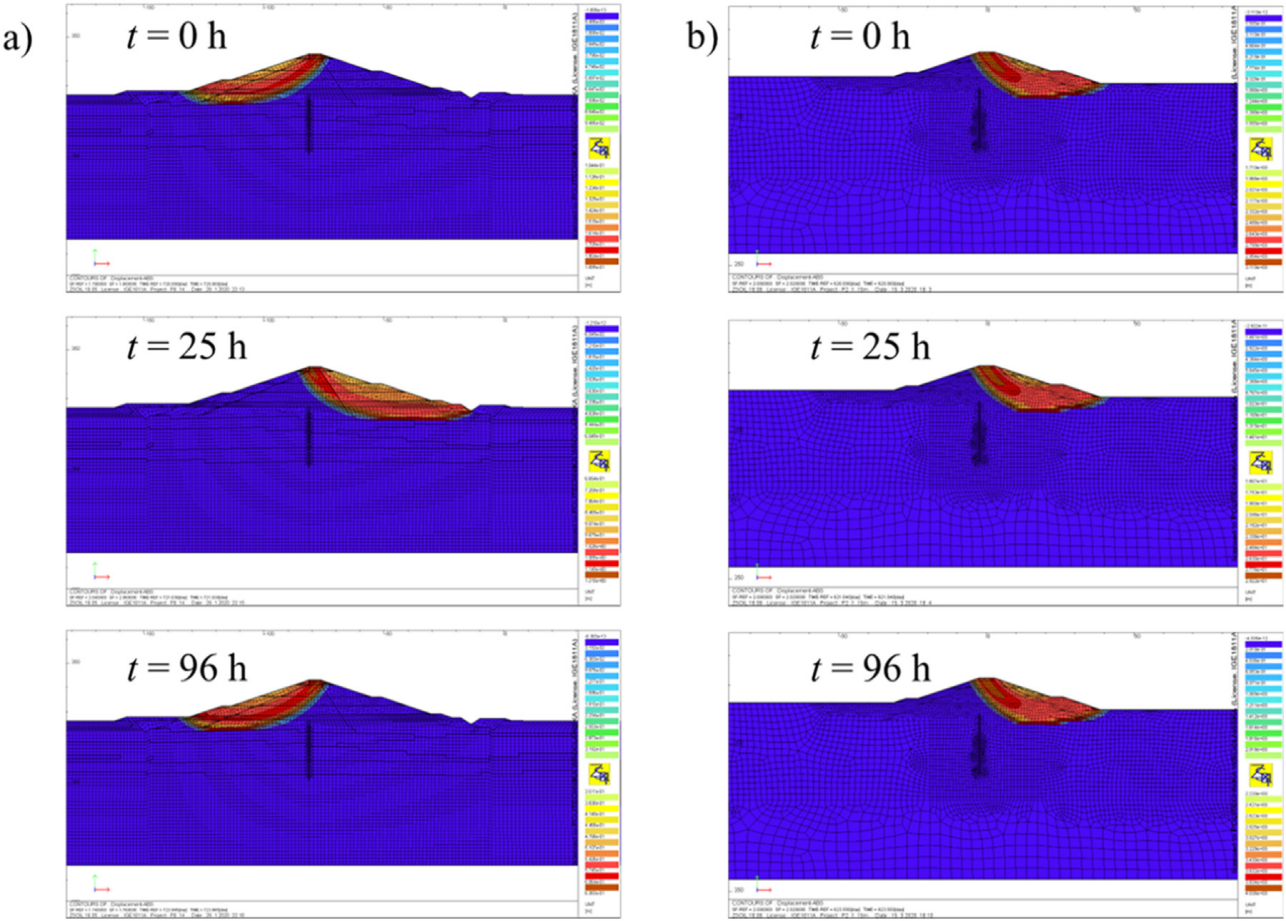
Potential failure mechanisms: a) Szalejów Górny, b) Krosnowice.

Due to the transient groundwater flow *F*(*t*) varies in time in the course of flood event. Thus, in order to evaluate the minimum value of *FOS* on which the design solutions depend, the stability analysis should be carried out at least a dozen times for selected moments in time of the analyzed process. The examples of *F*(*t*) changes with time are presented in Fig. 12. Note that for different objects considered variation in time is either apparently pronounced (as for Szalejów Górny reservoir – see Fig. 12a) or not present (see Fig. 12b related to Krosnowice reservoir). Fig. 13 shows the potential mechanisms of failure for different moments of time.

Assessments of the deformation as well as the stability are carried out for all the construction and operational phases, in particular after completion of the dam construction and during the damming process according to the assumptions made in the project. In the event of non-compliance with the limit states, changing the shape of the dam, a reinforcement of the subsoil or an increase of the depth of the barrier have to be considered. In such situations it is necessary to repeat the calculations described in Steps 2 and 3.

## Ethics statements

n.a.

## CRediT authorship contribution statement

**Maciej Sobótka:** Conceptualization, Methodology, Writing – original draft, Writing – review & editing, Visualization. **Adrian Różański:** Methodology, Writing – original draft, Writing – review & editing. **Jakub Rainer:** Investigation, Visualization, Writing – review & editing. **Mikołaj Masłowski:** Visualization, Writing – original draft.

## Declaration of Competing Interest

The authors declare that they have no known competing financial interests or personal relationships that could have appeared to influence the work reported in this paper.

## Data Availability

The data that has been used is confidential.
